# Marine Durability of Alkali-Activated Materials Under Multi-Ion Attack: Mechanisms, Responses, and Mitigation Strategies

**DOI:** 10.3390/ma19143058

**Published:** 2026-07-16

**Authors:** Xue Bai, Zhiliang Zhou, Menglei Yue, Lilin Yang, Tong Gao, Man Feng, Ning Xie

**Affiliations:** 1 Shandong Provincial Key Laboratory of Green and Intelligent Building Material, University of Jinan, Jinan 250022, China; 202321100342@stu.ujn.edu.cn (X.B.); yanglilin2019@163.com (L.Y.); fengman@stu.ujn.edu.cn (M.F.); mse_xien@ujn.edu.cn (N.X.); 2 Key Laboratory of Advanced Marine Materials, Institute of Oceanology, Chinese Academy of Sciences, Qingdao 266071, China; 3 Guizhou Provincial Architectural Design & Research Institute Co., Ltd., No. 28 Lincheng West Road, Guanshanhu District, Guiyang 550081, China; 4 School of Chemistry and Chemical Engineering, University of Jinan, Jinan 250022, China; gt1980614889@163.com

**Keywords:** alkali-activated materials (AAMs), marine durability, multi-ion attack, precursor–activator chemistry

## Abstract

Alkali-activated materials (AAMs) are widely regarded as promising alternatives to ordinary Portland cement for marine engineering because of their low carbon footprint, efficient utilization of industrial by-products, and potentially favorable mechanical and durability performance. However, their long-term application in marine environments remains challenging, as the original advantages of AAMs can be progressively weakened by the individual and coupled actions of aggressive seawater ions, particularly chloride (Cl^−^), sulfate (SO_4_^2−^), and magnesium (Mg^2+^). These ions affect AAMs through distinct but interconnected mechanisms, including chloride binding and transport, competitive ion interactions, phase transformation, destabilization of reaction products, pore-structure evolution, and the subsequent degradation of macroscopic properties. Meanwhile, the response of AAMs to marine exposure is highly system-dependent, since precursor chemistry, activator design, reaction-product assemblage, and pore structure strongly govern their resistance to ion attack. In recent years, considerable efforts have been devoted to improving the marine durability of AAMs through composition and phase design, pore-structure refinement, and transport control. Nevertheless, current understanding remains fragmented, particularly regarding the coupled effects of multiple seawater ions and the links between microstructural evolution and long-term performance. The primary purpose of this review is to provide a systematic overview of the marine durability of AAMs from the perspectives of multi-ion threats, material-dependent responses, and existing mitigation strategies. Particular emphasis is placed on the roles of Cl^−^, SO_4_^2−^, and Mg^2+^, the controlling effects of precursor and activator chemistry, and the translation of micro-mechanisms into macroscopic durability evolution. By integrating these aspects within a unified framework, this review aims to support the design and application of AAMs for reliable long-term use in coastal and offshore engineering. Future research should prioritize standardized multi-ion exposure protocols, coupled transport–reaction models, long-term field validation, and durability assessment of reinforced AAM concretes under realistic marine conditions.

## 1. Introduction

Alkali-activated materials (AAMs) have emerged as one of the most promising alternatives to ordinary Portland cement (OPC) owing to their low carbon footprint, broad raw material availability, and high compositional tunability [1,2]. In addition to these environmental advantages, AAMs can exhibit favorable mechanical performance, chemical resistance, and durability when properly designed [3,4]. These merits have promoted their application potential in a wide range of fields, including structural materials, transportation infrastructure, repair and strengthening systems, waste stabilization, and precast products. Among these emerging application scenarios, coastal and marine engineering has attracted particular attention, because it requires construction materials that are not only durable under aggressive exposure conditions but are also compatible with the growing demand for low-carbon development [5]. However, their use in marine environments remains challenging, wherein the real marine environments constitute a chemically complex exposure field characterized by the long-term coexistence of multiple aggressive ions, such as chloride (Cl^−^), sulfate (SO_4_^2−^), and magnesium (Mg^2+^). Chloride ions are widely recognized for their critical role in reinforcement corrosion and durability loss [6]. Sulfate ions can participate in competitive reactions and phase alteration, while magnesium ions may destabilize reaction products and significantly modify pore solution chemistry [7]. More importantly, these ions do not act independently under practical seawater exposure; instead, they interact simultaneously with the binder matrix and with each other, making the deterioration behavior of AAMs much more complex than that observed in conventional single-ion studies.

The complexity of marine durability in AAMs is further amplified by the intrinsic diversity of the materials themselves. Unlike OPC-based binders, AAMs do not represent a single chemistry, but rather a family of materials whose behavior is strongly governed by precursor composition and activator design. High-calcium systems generally form C-(A)-S-H-type gels and layered double hydroxides, low-calcium systems are typically dominated by N-A-S-H-type gels, and hybrid systems often contain mixed reaction products with transitional characteristics [8]. These differences in phase assemblage, pore solution chemistry, and pore structure strongly influence the way AAMs interact with marine ions, including ion binding, dissolution–precipitation behavior, phase stability, and transport resistance. In addition, parameters such as alkali concentration [9], activator modulus [9,10], alkali cation type [11], curing regime, and temperature [12] may further regulate these internal characteristics, resulting in highly system-dependent durability responses under marine exposure.

Therefore, the durability of AAMs cannot be adequately understood through a single-process perspective. Rather than being governed solely by chloride ingress or by the action of any one aggressive ion, it is controlled by the coupled reconfiguration of the internal equilibrium of the material under multi-ion seawater exposure. The ingress of Cl^−^, SO_4_^2−^, and Mg^2+^ may alter reaction products through adsorption, ion exchange, dissolution, precipitation, or phase transformation, while simultaneously modifying pore structure and transport pathways within the matrix. As a consequence, ion binding behavior [11], ion mobility, phase stability, and moisture transport become strongly interrelated. These microstructural and chemical changes are eventually manifested in macroscopic durability indicators [13], such as compressive strength, mass stability, porosity, water absorption, and long-term service performance. Representative studies have shown that marine-induced changes in AAMs can be quantitatively significant. For example, sulfate-containing aggressive exposure has been reported to cause approximately 25% compressive-strength loss in selected alkali-activated mortars [14]. In seawater-exposed metakaolin/slag geopolymers, the external solution pH was observed to increase from 9.14 to 11.00 and then gradually decrease to about 9.50, indicating the dynamic role of brucite precipitation and leaching [15]. Other studies have reported 3–5% mass loss while retaining 32.64 MPa residual compressive strength after short-term artificial seawater exposure [16], and an approximately 17% reduction in porosity under seawater curing relative to freshwater curing in selected AAM systems [17]. These results indicate that marine exposure may cause measurable strength loss, pH evolution, mass change, and pore-structure modification, while also showing that short-term densification and long-term deterioration may coexist depending on material chemistry and exposure condition. These results indicate that marine exposure may cause measurable strength loss, pH evolution, mass change, and pore-structure modification, while also showing that short-term densification and long-term deterioration may coexist depending on material chemistry and exposure condition.

Although substantial progress has been made in recent years, current knowledge remains fragmented. Many existing studies still focus primarily on chloride-related behavior or simplified exposure conditions, whereas the specific roles of sulfate and magnesium ions, as well as their coupled interactions with chloride, have not yet been systematically clarified. Furthermore, the relationships between precursor chemistry, reaction-product evolution, microstructural alteration, and long-term durability performance under marine multi-ion environments remain insufficiently established. In this context, this review provides a comprehensive overview of the durability of AAMs in marine environments, with particular emphasis on the roles of chloride, sulfate, and magnesium ions, as well as the controlling effects of precursor chemistry, activator system, phase assemblage, transport behavior, and hardened-state property evolution. It should also be noted that much of the existing understanding has been derived from simplified single-ion exposure studies, which are valuable for isolating specific mechanisms but insufficient for representing realistic marine environments. In particular, recent evidence shows that the chloride transport behavior observed under seawater exposure cannot be directly extrapolated from pure NaCl systems, because sulfate, magnesium, and leaching may alter chloride binding, phase stability, and transport pathways in a coupled manner [7,18].

This review aims to clarify how multi-ion marine exposure progressively weakens the protective functions of AAMs and why different AAM systems show divergent durability responses. Compared with previous reviews that mainly focus on general AAM durability, chloride transport, seawater mixing, or individual aggressive ions, this review emphasizes a coupled multi-ion perspective. Specifically, this review contributes in the following aspects:

- It establishes a coupled multi-ion perspective for understanding the marine degradation of AAMs by considering the interactive effects of Cl^−^, SO_4_^2−^, and Mg^2+^, rather than treating these ions as independent degradation factors.
- It introduces a chemical degradation pathway analysis that links ion ingress, pore solution evolution, chloride binding and release, sulfate-induced phase alteration, magnesium-induced alkalinity loss, gel decomposition, and pore-structure reorganization.
- It connects material-dependent durability responses with mitigation strategies and macroscopic performance indicators, thereby providing a more practical basis for comparing AAM systems under marine exposure.

Through this framework, the review aims to provide a critical and design-oriented synthesis for understanding and improving the long-term marine durability of AAMs.

## 2. Civil Engineering Background and Advantages of AAMs for Marine Engineering Applications

In this section, the civil engineering relevance and potential advantages of AAMs are discussed on the basis of documented findings from previous studies, rather than as general research objectives. AAMs have attracted increasing interest in marine engineering because they combine environmental benefits with a potentially favorable durability profile. Compared with OPC, AAMs can significantly reduce the consumption of clinker-based raw materials and enable the valorization of industrial by-products and solid wastes such as slag, fly ash, and metakaolin [1,2,3,4]. This low-carbon and resource-efficient nature makes AAMs particularly attractive for marine and coastal infrastructure, where construction volumes are often large and long-term sustainability has become an increasingly important design consideration.

From a civil engineering perspective, the potential application of AAMs should be discussed not only in terms of binder chemistry, but also in relation to real structural functions, exposure conditions, and durability verification at the component scale. Recent studies on cementitious composite systems for structural strengthening have emphasized that the practical implementation of advanced cement-based materials requires evaluation under service-relevant aggressive environments, rather than relying solely on initial mechanical performance. For example, Cascardi et al. [19] investigated glass FRCM-confined concrete cylinders exposed to simulated alkaline environments for 1000, 2000, and 3000 h, followed by axial compression testing and microscopic examination. Their results showed that prolonged chemical exposure progressively weakened the fiber–matrix interface and reduced confinement effectiveness, highlighting the importance of linking material degradation mechanisms with structural performance in civil engineering applications. Although the investigated system differs from AAM binders, this study provides a useful civil engineering reference framework for the present review: marine durability should be evaluated through the combined consideration of exposure realism, material degradation, interfacial stability, and component-level serviceability.

Beyond their sustainability advantages, AAMs also exhibit several material characteristics that make them promising candidates for aggressive marine exposure conditions. Many AAM systems are capable of developing relatively dense matrices and refined pore structures, which can reduce the ingress of external ions and moisture [20,21,22]. Depending on precursor chemistry, AAMs may also form reaction products with appreciable ion-binding capacity, including C-(A)-S-H-type gels, N-A-S-H-type gels, and layered double hydroxide phases [9,10,11,12,23,24]. These phases can affect the uptake, immobilization, or transport of aggressive ions, thereby influencing resistance to seawater attack. Moreover, AAMs have often shown good mechanical strength, satisfactory chemical resistance, and, in some systems, superior chloride resistance compared with OPC-based materials. For example, long-cured alkali-activated slag has been reported to outperform OPC in chloride binding because of its higher content of C-S-H/C-A-S-H gel and the development of a more negatively charged binding surface [23]. These findings indicate that selected AAM systems can exceed conventional binders in chloride-related resistance, although such advantages remain strongly dependent on precursor chemistry and exposure conditions. A more explicit quantitative comparison has also been reported in the literature: the 28-day chloride binding coefficient of a representative geopolymer system reached 83.372%, compared with 73.223% for OPC under the same curing condition, further highlighting the potential of selected alkali-activated systems to outperform conventional binders in chloride-related durability [25].

Because of these combined advantages, AAMs have shown potential in a wide range of marine-related applications, including structural and non-structural concrete elements, repair and overlay materials, protective layers, precast units, and other cementitious products exposed to seawater or marine aerosols [13,20,21,22]. Their tunability also makes them attractive for designing materials adapted to different marine scenarios, such as immersion zones, splash zones, tidal environments, and coastal atmospheric exposure. However, this application potential should not be interpreted as an indication that all AAMs are inherently suitable for marine use. Rather, AAMs provide a promising material platform whose final performance depends strongly on how effectively their internal composition and microstructure can resist the gradual degradation caused by marine ions.

## 3. Progressive Degradation of AAMs Under Marine Multi-Ion Environments

Before discussing the roles of individual marine ions and their coupled effects, the basis for evaluating the literature summarized in this section should be clarified. The information discussed here was not generated from new experimental tests by the authors but was extracted and synthesized from previously published studies. These studies adopted different exposure solutions, ion concentrations, specimen geometries, curing regimes, testing durations, and characterization methods. Therefore, the purpose of this section is not to rank all AAM systems using a single standardized test protocol, but to interpret degradation trends by comparing the types of evidence reported in the original studies.

Table 1 summarizes the main categories of evidence used to interpret the degradation behavior of AAMs under marine multi-ion exposure. Chloride-related indicators, such as diffusion or migration coefficients, passed charge, free/bound chloride content, and binding isotherms, are used to evaluate whether chloride mainly remains mobile or is immobilized within the matrix. Sulfate-related evidence, including sulfate immersion, expansion, strength loss, and gypsum or ettringite formation, is used to determine whether sulfate acts primarily as a competing ion, a pore-filling species, or a phase-damaging agent. Magnesium-related observations, such as pH evolution, brucite precipitation, C-(A)-S-H decomposition, and M-S-H formation, are used to identify alkalinity loss and gel destabilization. Multi-ion exposure data are then used to determine whether these individual effects are additive, competitive, or mutually amplified. Macroscopic durability indicators and microstructural/chemical characterization are interpreted together to connect chemical degradation with engineering-relevant performance.

This evidence-based structure guides the organization of Section 3.1, Section 3.2, Section 3.3 and Section 3.4. Chloride is first discussed as a transport- and binding-controlled threat, sulfate as a competing and phase-altering ion, magnesium as an alkalinity-reducing and gel-destabilizing ion, and finally multi-ion exposure as a coupled process that cannot be inferred from single-ion results alone. Through this structure, marine deterioration is treated as the progressive loss of chloride-binding capacity, transport resistance, and internal chemical stability under coupled seawater-ion exposure.

### 3.1. Chloride as the Primary Transport-Driven Threat

Among the major seawater ions, chloride is the most direct threat to the long-term durability of AAMs because it is closely associated with ingress, accumulation, and, in reinforced systems, corrosion risk. Once chloride enters the matrix, part of it may be immobilized through chemical binding or physical adsorption, whereas the remaining free chloride can continue to migrate through the pore network. Therefore, the actual threat posed by chloride depends not only on how much chloride enters the material, but also on how effectively the material can bind it and how rapidly the unbound fraction can be transported inward.

Pore structure, phase assemblage, and pore solution chemistry in AAMs are strongly governed by precursor type. As hydration products continue to form, they gradually occupy the spaces originally filled by water and air, resulting in a densified microstructure that impedes chloride ingress [26]. In high-calcium systems such as AAS, C-(A)-S-H is the dominant phase and the matrix generally exhibits relatively low chloride diffusion coefficients, whereas low-calcium fly ash-based geopolymer concrete often shows lower chloride diffusion resistance and chloride-binding capacity [27]. This contrast reflects the broader classification-related differences in reaction products and binding mechanisms that are discussed in Section 4.

Several studies show how transport resistance can be improved or weakened by microstructural design. Behfarnia and Rostami [28] reported that microsilica densified alkali-activated slag concrete and improved chloride resistance, whereas nanosilica tended to agglomerate and generate localized heterogeneity that increased chloride transport. Bernal et al. [20] found that in slag–metakaolin blends, increasing metakaolin content and activator concentration generally reduced water sorptivity and chloride permeability, but they also noted that RCPT does not directly quantify chloride diffusion in AAMs. Liu et al. [29] further showed that MgO, Mg-Al-NO_3_ LDH, and calcined LDH-CO_3_ could all improve the chloride resistance of fly ash–slag blends, with calcined LDH delivering the greatest enhancement in chloride-binding capacity.

Activator chemistry is another major control on chloride transport. Silicate-based activators usually provide better chloride ingress resistance than hydroxide-based activators because additional soluble silica promotes gel formation and matrix densification [30]. Hu et al. [22] demonstrated that increasing silicate modulus and alkali dosage enhanced strength, refined pore structure, and reduced both the chloride migration coefficient and the passed charge in rapid permeability tests, whereas higher fly ash content tended to coarsen the pore structure and increase chloride transport. Ravikumar and Neithalath [21] likewise showed that critical pore size can exert a stronger control on chloride transport than total porosity. By contrast, sodium carbonate-activated slag tends to develop a more open pore structure and lower chloride-binding capacity because of its lower alkalinity and slower slag dissolution [31].

Single-ion chloride exposure studies remain essential for clarifying binding behavior and transport kinetics in relatively controlled systems [32]. However, they cannot capture the additional effects introduced by sulfate-related competition or magnesium-induced phase destabilization under realistic seawater exposure [7,18].

### 3.2. Sulfate as a Competitor and Phase-Altering Ion

Compared with chloride, sulfate affects AAMs in a less direct but chemically more complex manner. Sulfate ions may compete with chloride for reactive sites or layered phases, alter the stability of existing products, and induce secondary phase formation. As a result, sulfate can modify both chloride binding and chloride transport, even when chloride remains the primary ion of concern from a service-life perspective.

Recent studies reveal that sulfate plays a dual role in chloride transport. Recent studies reveal a dual role of sulfate in chloride transport. Combined sulfate–chloride exposure can cause ~25% compressive strength loss due to gypsum formation and C-A-S-H degradation, which creates additional ingress pathways [14]. Sulfate also competes with chloride for binding sites on C-N-A-S-H gels, reducing chemically bound chloride, though associated gel expansion may partially densify the matrix [33].

Under dry–wet cycles, early ettringite and gypsum formation temporarily fills pores and reduces diffusivity, but later excessive expansion generates microcracks that accelerate chloride penetration [34]. In low-calcium systems, limited calcium availability prevents expansive products, so sulfate mainly competes for adsorption sites with only moderate effects on diffusion [35]. Another study [7] further showed that the effect of sulfate on chloride transport depends strongly on the accompanying cation: Na_2_SO_4_ + NaCl could lead to long-term densification, whereas MgSO_4_ + NaCl destabilized Friedel’s salt, increased free chloride concentration, and accelerated transport.

**Figure 1:**
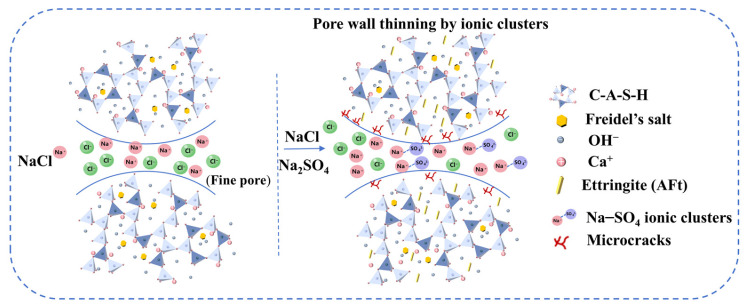
Divergent mechanisms governing pore wall–sulfate interactions in alkali-activated slag under chloride–sulfate diffusion. Redrawn by the authors based on mechanisms and experimental observations reported in Ref. [7].

Figure 1 summarizes the sulfate-related pore-wall interaction mechanisms reported for alkali-activated slag under chloride–sulfate diffusion. The figure is included to clarify the dual role of sulfate: depending on phase assemblage and exposure stage, sulfate may contribute to local pore filling and temporarily reduce diffusivity, or it may promote phase alteration, expansion damage, and microcrack-assisted transport. This distinction supports the view that sulfate effects should be interpreted dynamically rather than as a uniformly beneficial or detrimental process.

These observations indicate that sulfate-related deterioration is not limited to competitive chloride binding. Once sulfate-induced phase alteration exceeds the beneficial effect of temporary pore filling, microcrack formation and loss of structural continuity may become dominant, thereby converting a chemically modified matrix into a mechanically weakened and more transport-prone one. Therefore, the available evidence suggests that sulfate-related effects are not merely qualitative. Under coupled sulfate–chloride conditions, compressive strength loss can reach approximately 25% in some alkali-activated mortars [14], while the transport response may shift from temporary pore filling and reduced diffusivity to microcrack-assisted chloride ingress as exposure proceeds [34].

A critical distinction should therefore be made between single-ion and multi-ion exposure studies. Single-ion sulfate studies are useful for identifying phase alteration, competitive binding, and transient pore-filling effects in a relatively simplified environment. By contrast, multi-ion studies demonstrate that these sulfate-related effects may be substantially modified once magnesium and chloride coexist. Chen and Ye [7] showed that although sulfate can suppress chloride transport in some scenarios, the simultaneous presence of magnesium may overwhelm this suppression by lowering alkalinity and releasing previously bound chloride. Liu et al. [18] further confirmed that in high-concentration chloride–sulfate–magnesium solutions, degradation cannot be interpreted as the sum of separate ionic effects, because magnesium-induced decomposition of reaction products and chloride release fundamentally changes the transport process.

### 3.3. Magnesium as the Most Destabilizing Agent

If chloride mainly probes transport resistance and sulfate perturbs binding and phase relations, magnesium often acts as the ion that most fundamentally destabilizes the internal chemical and structural state of AAMs. The entry of Mg^2+^ into the matrix is associated with the consumption of hydroxyl ions and the precipitation of Mg(OH)_2_ (brucite), which lowers local alkalinity and undermines the stability of many AAM reaction products.

Jiang et al. [32] showed that under a specific short-term exposure condition, the apparent chloride diffusion coefficient of AAS mortar decreased in the order KCl > NaCl > MgCl_2_ > CaCl_2_, suggesting that Mg^2+^ may initially retard transport because Friedel’s salt formation and pore filling locally densify the matrix. However, long-term and more realistic marine exposures tell a different story. Cai and Liu [36] emphasized that Mg-containing solutions are generally more damaging than sodium sulfate solutions because magnesium participates directly in phase transformation. Chen and Ye [7] demonstrated that the aggravating effect of magnesium on chloride diffusion overwhelms the suppressing effect of sulfate; magnesium-induced pH reduction, brucite formation, partial dissolution of C-A-S-H, and pore coarsening together accelerate chloride ingress.

Liu et al. [18] further showed, using high-concentration chloride–sulfate–magnesium multi-salt solutions, that Mg^2+^ promotes chloride diffusion by inducing C-A-S-H decomposition and M-S-H formation, while simultaneously altering binding phases and releasing previously bound chloride. Thus, Mg^2+^ is particularly critical because it does not merely add another ionic stress; it dismantles the internal alkaline and phase equilibrium that originally supported chemical stability and transport resistance, thereby promoting pore coarsening, phase destabilization, and the progressive weakening of the solid skeleton.

The quantitative significance of magnesium-related degradation is also evident from literature-reported pH evolution during seawater exposure. For instance, the external solution pH of metakaolin/slag geopolymers was reported to increase from 9.14 to 11.00 and then gradually decrease to about 9.50 as brucite accumulated [15]. Such evidence confirms that Mg^2+^ does not simply coexist with chloride and sulfate, but actively reshapes the chemical conditions governing long-term durability.

### 3.4. Multi-Ion Coupling: From Separate Threats to Progressive Loss of Protections

The real severity of marine environments lies in the fact that chloride, sulfate, and magnesium do not act independently. A critical comparison between single-ion and multi-ion studies shows that the latter cannot be interpreted as a simple superposition of the former. Single-ion chloride studies are useful for isolating chloride binding and transport resistance; single-ion sulfate studies reveal competitive binding, phase alteration, and transient pore-filling effects; and magnesium-related studies highlight alkalinity reduction and decomposition of reaction products. However, under coupled marine exposure, these mechanisms interact dynamically. Sulfate may temporarily reduce chloride diffusivity through pore filling or matrix densification, whereas magnesium can offset this effect by destabilizing chloride-binding phases, lowering pH, and reopening transport-effective pore pathways [7,18]. As a result, durability trends derived from single-ion exposure may be reversed or amplified in multi-ion environments. To make this distinction explicit, Table 2 compares the mechanistic value and limitations of representative single-ion and multi-ion exposure studies.

These comparisons also indicate that differences among published results are not merely experimental scatter. They are closely related to precursor calcium content, activator type, alkali concentration, solution chemistry, exposure duration, wetting–drying regime, and the indicator selected for evaluation. As a result, direct ranking of Cl^−^, SO_4_^2−^, and Mg^2+^ effects are meaningful only when the material system and exposure protocol are clearly specified. Future studies should therefore move from isolated single-ion tests toward benchmark multi-ion exposure protocols that combine transport indicators, phase characterization, pore-structure analysis, and macroscopic performance retention.

Overall, the available literature suggests several consensuses and remaining inconsistencies. A general consensus is that chloride-related degradation is primarily transport- and binding-controlled, sulfate-related effects are strongly dependent on phase assemblage and exposure stage, and magnesium-containing environments are often more chemically destabilizing than sodium-based chloride or sulfate solutions. However, apparently conflicting results also exist. For example, some short-term Mg-containing exposures report reduced chloride diffusivity or improved compactness, whereas longer-term or coupled multi-salt studies show accelerated chloride transport, bound-chloride release, and pore coarsening [7,18]. These discrepancies are not merely experimental scatter; they are related to precursor calcium content, activator type, solution chemistry, exposure duration, wetting–drying regime, and the durability indicator selected for evaluation. Therefore, direct ranking of Cl^−^, SO_4_^2−^, and Mg^2+^ effects is meaningful only when the material system and exposure protocol are clearly specified.

**Figure 2:**
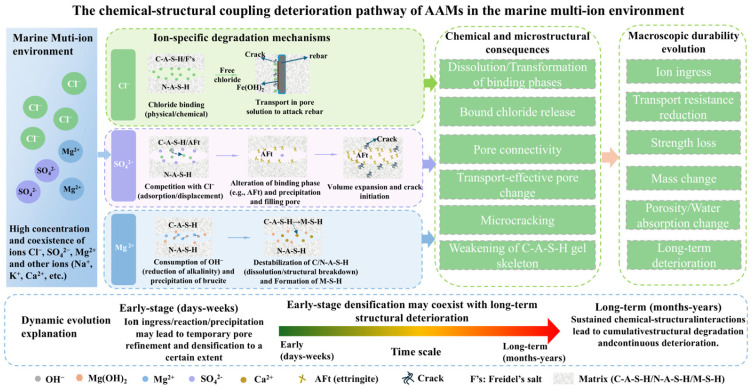
Schematic summary of coupled multi-ion interaction mechanisms in AAMs under marine exposure, distinguishing ingress pathways, chemical/phase-related effects, and microstructural evolution under Cl^−^–SO_4_^2−^–Mg^2+^ exposure. Prepared by the authors based on mechanisms reported in Refs. [7,9,10,11,12,14,15,18,24,33,34,35,39,40].

To integrate the ion-specific discussion above, Figure 2 summarizes the coupled multi-ion interaction mechanisms in AAMs under marine exposure. The diagram distinguishes three levels of interaction: ion transport effects, including ion ingress through connected pores and wetting–drying-assisted redistribution; chemical/phase-related effects, including chloride binding/release, sulfate competition, brucite precipitation, and C-(A)-S-H destabilization; and microstructural evolution, including pore filling, pore coarsening, microcracking, and reopening of transport pathways. This schematic provides the transition from the ion-specific discussion in Section 3 to the chemical degradation pathway analysis in Section 4.

## 4. Chemical Degradation Pathway Analysis Under Marine Multi-Ion Exposure

The preceding section shows that single-ion findings cannot be directly extrapolated to realistic multi-ion marine exposure. To further clarify the underlying degradation sequence, this chapter reorganizes the reported Cl^−^, SO_4_^2−^, and Mg^2+^ effects into a chemical pathway framework. In this framework, marine deterioration is interpreted as a sequence of linked processes: external ion ingress → pore solution modification → ion binding, exchange, dissolution, or precipitation → phase transformation and destabilization → pore-structure reorganization → macroscopic durability degradation. This pathway-based interpretation is consistent with recent studies showing that seawater-induced chloride transport in AAMs cannot be extrapolated directly from pure NaCl exposure, because sulfate, magnesium, and leaching can simultaneously alter chloride binding, phase stability, and pore connectivity [7,18].

The first pathway is related to chloride ingress and binding. After entering the pore solution, Cl^−^ may remain as free chloride or be immobilized through physical adsorption, ion exchange, or chemical binding by C-(A)-S-H-type gels, N-A-S-H-type gels, AFm-related phases, and LDH-type phases [9,10,11,12,24,39,40,41]. The effectiveness of this pathway is strongly controlled by precursor chemistry and activator design. In AAS systems, chloride binding is closely related to the formation and surface properties of C-(A)-S-H, AFm, and hydrotalcite-like phases [11,24,39,41], whereas in low-calcium fly ash or metakaolin systems, chloride immobilization is more dependent on adsorption by N-A-S-H-type gels or chloride-bearing zeolitic phases [37,40,42]. For example, seawater-mixed alkali-activated slag and fly ash systems have been shown to form different chloride-bearing phases, indicating that chloride immobilization pathways vary with precursor chemistry [37]. Therefore, chloride ingress does not immediately imply degradation if a stable binding pathway is maintained. Degradation begins when the bound chloride reservoir is destabilized by changes in pH, sulfate competition, magnesium attack, or phase dissolution, which increases the free chloride fraction and enhances the risk of further transport and reinforcement corrosion [7,18].

The second pathway is associated with sulfate-induced competition and phase alteration. SO_4_^2−^ can compete with Cl^−^ for binding sites or layered phases and may promote the formation of sulfate-bearing products [7,33,34,35]. In some AAM systems, limited sulfate reaction may temporarily densify the matrix by pore filling, thereby reducing ion mobility [35]. However, when sulfate-induced phase alteration becomes excessive, gypsum- or ettringite-related expansion, gel destabilization, and microcrack formation may occur [14,34,43]. This transforms sulfate from a chemically competitive ion into a structurally damaging species. The consequence is the opening of new transport pathways, which can accelerate chloride ingress and amplify subsequent magnesium-related destabilization [7,18,34].

The third and often most destructive pathway is magnesium-induced alkalinity reduction and gel decomposition. Mg^2+^ can react with hydroxyl ions to form brucite, reducing the alkalinity of the local pore solution [7,15,43]. This pH reduction weakens the stability of calcium-bearing reaction products such as C-(A)-S-H [7,18]. As the calcium-bearing gel decomposes or decalcifies, Mg-bearing silicate or aluminosilicate hydrates such as M-S-H or M-A-S-H may form [7,18,43]. This pathway is particularly harmful because it simultaneously consumes alkalinity, destroys the binding phases responsible for chloride immobilization, weakens the load-bearing gel network, and coarsens the pore structure [7,28]. As a result, magnesium attack can convert an initially dense and chemically protective AAM matrix into a more porous, less alkaline, and less mechanically stable material.

Under realistic seawater exposure, these pathways occur simultaneously and interact with one another. Sulfate may initially reduce chloride mobility through pore filling or competition, but magnesium can reverse this apparent benefit by destabilizing chloride-binding phases and lowering the pore solution pH [7,18]. Once C-(A)-S-H, AFm, or LDH-related phases are altered, previously bound chloride may be released, increasing the free chloride concentration in the pore solution [7,18,24,44]. At the same time, brucite precipitation, M-S-H/M-A-S-H formation, and gel decomposition reorganize the pore structure and reopen transport-effective pathways [7,18,43]. Therefore, the chemical degradation pathway under marine exposure is not a linear process controlled by a single ion, but a coupled chain reaction involving ion ingress, binding competition, pH evolution, phase destabilization, chloride release, pore coarsening, and macroscopic property loss.

This pathway-based interpretation helps explain why some AAMs show short-term densification or strength retention under seawater exposure, whereas others exhibit long-term deterioration. In the early stage, continued reaction, chloride binding, sulfate-related pore filling, or brucite precipitation may temporarily reduce porosity or ion mobility [23,34]. In the later stage, however, accumulated chemical imbalance, magnesium-induced gel decomposition, sulfate-related cracking, and release of bound chloride may dominate [7,18,34]. Therefore, marine durability should be evaluated dynamically by considering whether the initial protective pathways can remain stable under combined ionic attack. Therefore, the critical issue under seawater exposure is not the presence of any single aggressive ion, but whether sulfate- and magnesium-induced chemical changes undermine the chloride-binding and transport-resisting functions of the AAM matrix. This pathway-based view explains why early densification or strength retention may be followed by long-term deterioration when binding phases are destabilized and transport-effective pores are reopened.

## 5. Material Basis of the Diverse Marine Durability Responses of AAMs

As shown in Table 2, single-ion studies are valuable for isolating specific processes such as chloride binding, sulfate-induced phase alteration, or magnesium-related alkalinity reduction. However, multi-ion exposure studies reveal coupled effects that cannot be inferred by simple superposition, particularly sulfate–chloride competition, magnesium-induced release of bound chloride, and the reopening of transport-effective pore pathways.

The highly variable marine durability of AAMs does not arise solely from differences in external exposure conditions. More fundamentally, it reflects the intrinsic diversity of AAM systems themselves. Unlike OPC-based binders, AAMs comprise a broad family of materials with markedly different precursor compositions, activator chemistries, reaction products, pore solution characteristics, and microstructures. These internal differences determine how marine ions are bound, transported, or allowed to destabilize the matrix.

### 5.1. Precursor Chemistry: High-Calcium, Low-Calcium, and Hybrid Systems

One of the most fundamental factors controlling marine durability in AAMs is precursor chemistry, particularly calcium content. From this perspective, AAMs can be broadly categorized into three groups: high-calcium systems, typically represented by slag-based binders; low-calcium systems, commonly based on fly ash or metakaolin; and hybrid systems, produced by blending high- and low-calcium precursors. These three categories differ not only in their dominant reaction products, but also in their chloride-binding mechanisms, pore structures, and phase stability under marine exposure.

High-calcium AAMs generally show strong chloride-binding capacity. High-calcium AAMs generally show strong chloride-binding capacity. For example, long-cured alkali-activated slag outperforms OPC due to higher C-S-H/C-A-S-H content and a more negatively charged surface [23]. Adding Ca(OH)_2_ can further promote Friedel’s salt formation, albeit with some strength reduction [45].

Low-calcium systems rely more on physical adsorption; chloride binding increases as slag content decreases in fly ash/slag blends due to greater contribution of N-C-A-S-H gels [46]. It has been further shown that Na-based fly ash–metakaolin geopolymers generally had higher chloride-binding capacity than K-based ones, and that some compositions could approach or exceed the chloride binding of OPC pastes [42]. Accordingly, low-calcium systems are often less reliant on chemical chloride fixation and may show more limited resistance to chloride ingress unless transport pathways are effectively refined through mixture design and activator optimization.

**Figure 3:**
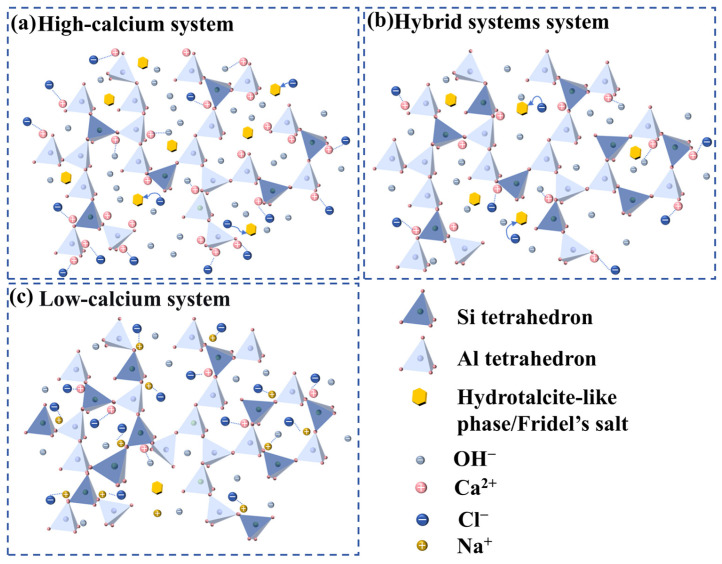
Schematic illustration of chloride-binding mechanisms for (**a**) high-calcium systems, (**b**) hybrid systems, and (**c**) low-calcium systems.

Hybrid systems frequently exhibit the most complex response. Ismail et al. [47] demonstrated that slag/fly ash blending changes the dominant gel assemblage depending on replacement level, moving from C-A-S-H-rich systems to mixed N-A-S-H/C-A-S-H systems as fly ash content rises. A study [48] showed that in seawater-mixed slag/fly ash AAMs, bound chloride increased by 3.78–17.42% with increasing fly ash dosage; when fly ash content was below 20%, chloride immobilization was mainly achieved by AFm-related chemical binding and adsorption on C-A-S-H, whereas at higher fly ash levels physical adsorption by (C,N)-A-S-H gels dominated. Consistent with the present study [48], the effects of slag/fly ash ratio on the chloride-binding capacity of alkali-activated slag/fly ash cements (AACs) were examined using binding isotherms at NaCl concentrations between 0.5 and 3.0 mol/L [9]. The results indicate that the Langmuir isotherm offers a superior description of chloride adsorption for most AACs. Across these hybrid systems, physical adsorption was often more important than chemical binding, and the adsorption capacity followed the order N-C-A-S-H > C-A-S-H > C-S-H. Representative chloride-binding demonstration for high-calcium, low-calcium, and hybrid systems are presented in Figure 3. This makes hybrid systems particularly important in practice, because they can combine the transport resistance of calcium-rich systems with the adsorption-related benefits of low-calcium gels, although their actual performance depends strongly on precursor ratio and exposure condition.

For clarity, the main categories of AAM systems and their representative marine-durability features are summarized in Table 3. As shown in the table, precursor calcium content governs not only the dominant gel assemblage, but also the balance among chemical chloride fixation, physical adsorption, transport resistance, and phase stability under marine exposure.

### 5.2. Activator System and Pore Solution Chemistry

If precursor chemistry defines the raw material basis of AAMs, the activator system determines how that basis is translated into a functional binder structure. Parameters such as alkali concentration, silicate modulus, alkali cation type, and activator composition strongly affect precursor dissolution, reaction extent, gel polymerization, and the chemistry of the pore solution.

**Figure 4:**
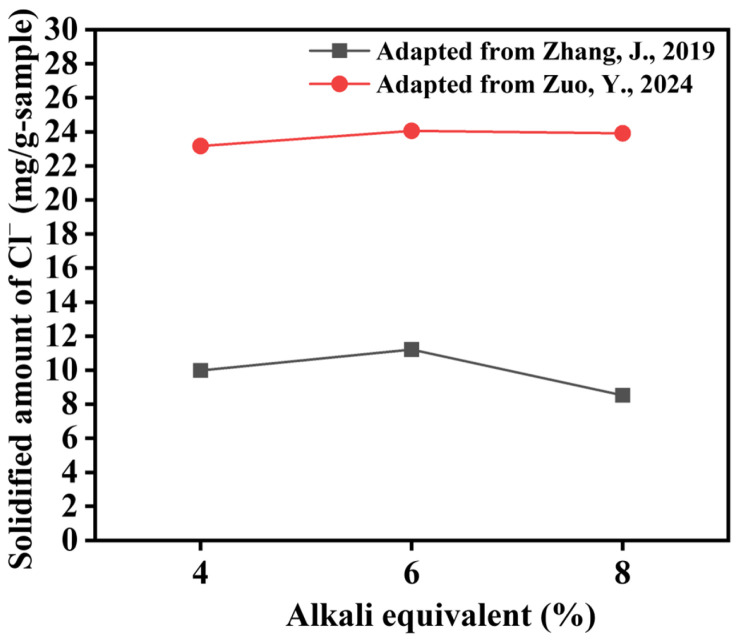
Total solidification of chloride ions for alkali-activated slag mixed with 3 wt.% NaCl solution. Redrawn based on the data reported in Refs. [9,49].

**Figure 5:**
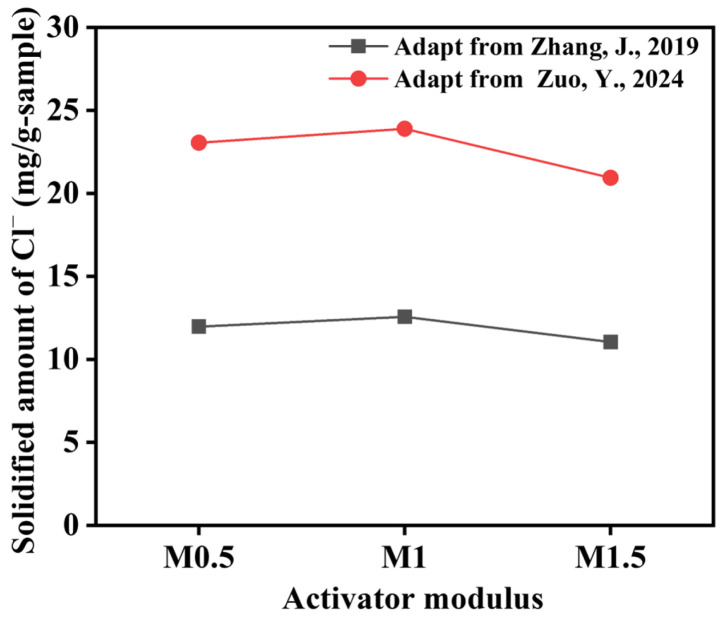
Simulated phase assemblage of alkali-activated slag with different alkali equivalent and activator modulus. Redrawn based on the thermodynamic simulation results reported in Refs. [9,49].

Alkali concentration can exert non-monotonic effects. Alkali concentration exerts non-monotonic effects. At low chloride concentrations, higher alkali content reduces bound chloride, whereas at high chloride concentrations, an intermediate alkali level (e.g., 6% Na_2_O) maximizes binding [9]. Increasing alkali concentration from 4% to 6% enhances both physical and chemical binding, but a further increase to 8% reduces physical binding due to higher pH and less C-(N)-A-S-H formation [49]. The effects of alkali concentration, activator modulus, and pH on chloride binding are illustrated in Figure 4 and Figure 5 and Table 4, which were used to connect measured chloride solidification behavior with simulated pore solution. Figure 4 and Figure 5 explain the simulated phase assemblage of alkali-activated slag with different alkali equivalent and activator modulus. Meanwhile, the bound chloride content of waterglass-activated (WAS) and NaOH-activated slag (NAS) at varying pH values is exhibited in Table 4. Thus, these two figures support the argument that chloride resistance in AAMs is governed by the combined effect of pore solution chemistry and binding-phase availability, rather than by chloride concentration alone.

The silicate modulus also changes chloride-binding behavior through gel chemistry. Zhang et al. [9] reported that increasing activator modulus enhanced chloride binding in slag-rich systems but reduced it in fly ash-rich systems. Zuo et al. [10,49] explained this by showing that higher soluble silica can promote gel polymerization and adsorption in some systems while simultaneously consuming dissolved Al that would otherwise contribute to Friedel’s salt formation.

Activator type and pH are equally important. Ye et al. [11] found that the binding capacity of AAS pastes depended strongly on activator composition, with AFm and hydrotalcite contributing 40–70% of total bound chloride. Fu et al. [42] showed that alkali cation type significantly influenced chloride binding in metakaolin-based geopolymers, with sodium-based systems generally outperforming potassium-based ones. Zhu et al. [12] reported that raising pH from 11 to 13.5 reduced bound chloride content in AAS, while Jain et al. [50] highlighted the competitive role of OH^−^ for binding sites in cementitious hydrates.

### 5.3. Phase Assemblage as the Immediate Carrier of Resistance

The influence of precursor and activator chemistry ultimately manifests in the reaction products formed within the hardened matrix. These products constitute the immediate internal carriers of marine resistance because they are the phases that directly interact with external ions.

**Figure 6:**
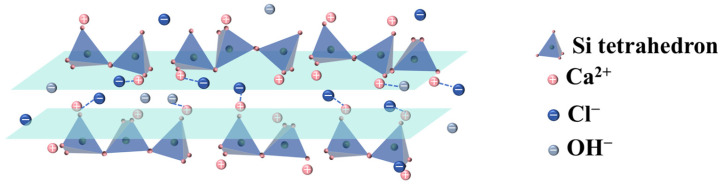
Schematic representation of chloride binding onto C-S-H gels. Modified and redrawn based on the chloride-binding mechanism reported in Ref. [39].

For C-S-H-type phases, chloride uptake depends on composition and surface properties. Zibara et al. [51] and Cai et al. [39] showed that higher Ca/Si ratios generally increase chloride-binding capacity because of larger specific surface area, more favorable surface potential, and more abundant adsorption sites. For C-A-S-H, the role of Al is more complex: Cai et al. [39] found that increasing Ca/Si and Al/Si improved chloride binding, while Yoshida et al. [52] predicted that Al incorporation can suppress chloride adsorption at constant Ca/(Si+Al) because of changes in site density. Wang et al. [53] also linked Al in C-A-S-H to higher chloride binding in blended cements through enhancement of Friedel’s salt formation. Figure 6 illustrates the chloride-binding mechanism associated with C-S-H-type gels. In calcium-bearing gel systems, chloride immobilization can occur through surface adsorption and interactions with calcium-rich sites. This mechanism is particularly relevant to high-calcium AAMs, where C-(A)-S-H-type products dominate the matrix and contribute to chloride retention.

**Figure 7:**
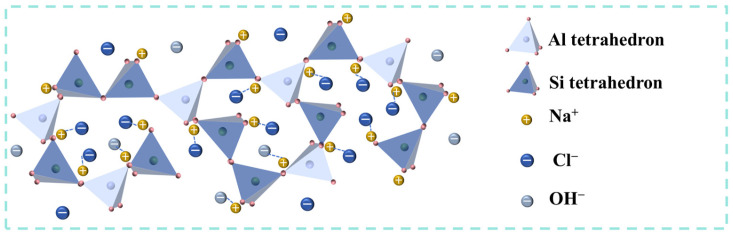
Schematic representation of chloride binding onto N-A-S-H gels. Modified and redrawn based on the chloride-adsorption mechanism reported in Ref. [40].

**Figure 8:**
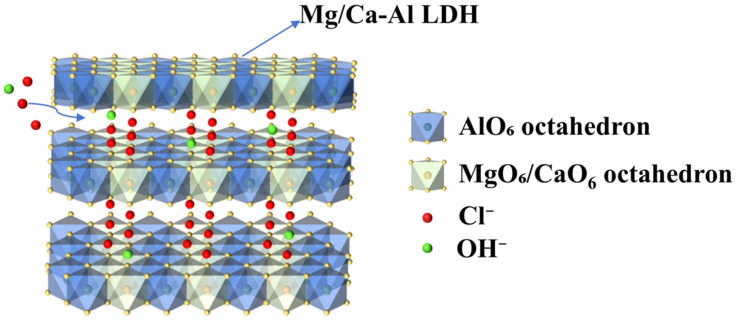
Schematic representation of chloride binding onto LDH-related phases. Modified and redrawn based on the chloride-uptake mechanism reported in Ref. [24].

N-A-S-H gels behave differently. Because they carry a net negative charge under alkaline conditions, their chloride binding is largely controlled by adsorption and by the balance between surface charge density and cation compensation. Wu [40] and related synthetic-gel studies indicate that lower Si/Al ratios generally increase chloride binding in N-A-S-H, which is consistent with more negative zeta potential and higher Na^+^ adsorption. Layered double hydroxides represent another important carrier. Ke et al. [24] showed that Mg-Al LDH and Ca-Al LDH can efficiently take up chloride under highly alkaline conditions, with surface adsorption dominating in Mg-Al LDH and interlayer/lattice substitution being more important in Ca-Al phases. Figure 7 and Figure 8 further show that chloride immobilization in AAMs is not limited to C-S-H-type gels. Figure 7 illustrates chloride adsorption associated with N-A-S-H-type gels, where chloride retention is mainly related to surface adsorption and charge-compensation effects. Figure 8 highlights chloride uptake by LDH-related phases, which can immobilize chloride through surface adsorption and interlayer anion exchange. Together, these two figures indicate that low-calcium aluminosilicate gels and layered phases may also contribute to chloride retention, although their binding mechanisms differ from those of calcium-bearing C-(A)-S-H-type products. Furthermore, Gluth et al. [41] showed that the apparent chloride uptake of LDH phases depends strongly on liquid-to-solid ratio, which partly explains discrepancies between practical and theoretical binding capacities.

## 6. Existing Mitigation Strategies Against Marine-Ion Threats in AAMs

Given that marine deterioration in AAMs originates from the progressive weakening of ion-binding capacity, transport resistance, and internal chemical stability, existing mitigation strategies have generally focused on reinforcing one or more of these protective functions. Most studies can be broadly grouped into two main routes: (i) composition and phase design, which aims to optimize precursor chemistry and reaction products to improve ion immobilization and phase stability; and (ii) pore structure and transport control, which seeks to reduce the accessibility and mobility of aggressive ions by refining the internal pore network. A third line of effort concerns more realistic exposure and evaluation strategies to determine whether these measures remain effective under actual marine conditions.

### 6.1. Composition and Phase Design

One direct strategy is to modify the chemical composition of AAMs so that the resulting reaction products are better suited to resist ion attack. Adjusting precursor blending allows researchers to regulate the relative amounts of C-(A)-S-H-, N-A-S-H-, LDH-, and hybrid-type products. Increasing calcium-bearing components can enhance chloride immobilization by promoting Friedel’s salt and AFm-related binding phases, but the trade-off with strength or long-term stability must be considered, as shown by the Ca(OH)_2_-modified AAS system discussed above.

Another important route is the targeted promotion of favorable phases. Ye et al. [11] and Ke et al. [24] highlighted the importance of AFm- and LDH-type phases for chloride uptake. Liu et al. [29] further showed that externally added Mg-Al LDH, MgO, and calcined LDH-CO_3_ can markedly improve chloride resistance in fly ash–slag blends, with calcined LDH delivering the greatest enhancement. Such results indicate that deliberate phase engineering can strengthen the chemical defense system of AAMs by improving both chloride-binding capacity and resistance to phase instability.

Despite these advantages, composition and phase design strategies are not universally beneficial. Increasing calcium-bearing phases may improve chloride immobilization by promoting Friedel’s salt and AFm-related binding, but may also reduce compressive strength or increase the long-term vulnerability of calcium-rich phases under magnesium-containing marine environments [45]. Similarly, externally added LDH-based phases can markedly enhance chloride binding [29], yet most current evidence is still derived from laboratory-scale chloride-focused studies, and their long-term stability under multi-ion coupling exposure remains insufficiently validated [7,18]. Therefore, the effectiveness of composition-based strategies should be judged not only by initial chloride-binding improvement, but also by their long-term phase stability and structural compatibility under realistic marine conditions.

### 6.2. Pore Structure and Transport Control

Many mitigation strategies target the physical ingress and migration of aggressive ions. Mixture design variables such as precursor fineness, water-to-binder ratio, activator dosage, silicate modulus, and curing regime are frequently adjusted to promote a denser microstructure with lower connectivity and more tortuous transport pathways. The desired outcome is not simply lower total porosity, but a reduction in the proportion of transport-effective pores and an improvement in the continuity of the solid skeleton.

Hu et al. [22] showed that increasing silicate modulus and alkali dosage refined pore structure and improved resistance to chloride transport, whereas high fly ash content coarsened the pore structure and reduced strength. Ravikumar and Neithalath [21] demonstrated that critical pore size exerts stronger control on chloride ingress than total porosity, underscoring the need to target transport-relevant pores rather than porosity alone. Behfarnia and Rostami [28] further showed that not all silica additions are equally beneficial: microsilica improved permeability resistance, whereas nanosilica agglomeration could increase heterogeneity and transport.

Pore-structure and transport-control strategies also involve clear trade-offs. For example, higher silicate modulus and alkali dosage may refine pore structure and reduce chloride transport, but excessive fly ash incorporation may coarsen the pore system and reduce strength [22]. In addition, not all densification measures are equally effective: microsilica can improve permeability resistance, whereas nanosilica agglomeration may increase heterogeneity and transport [28]. More importantly, a lower apparent transport coefficient at early stages does not necessarily imply durable long-term performance, because transport resistance may later be undermined by magnesium-induced phase destabilization, pore reopening, or cracking under coupled marine exposure [7,18]. Thus, transport-control strategies should be assessed dynamically rather than solely on the basis of short-term chloride migration or permeability data.

### 6.3. Exposure-Oriented Design and Evaluation

Many strategies that appear effective under simplified laboratory exposure may not retain the same benefit under realistic marine conditions. This is because real seawater involves coupled chloride, sulfate, magnesium, leaching, and often wetting–drying actions, whereas many available mitigation studies still rely on single-salt exposure, short-term immersion, or idealized transport tests [20,22]. As a result, an apparent improvement in chloride binding or transport resistance under controlled laboratory conditions does not necessarily translate into robust long-term performance in practical marine service. Consequently, researchers increasingly recognize that marine durability is not only a matter of material design, but also of exposure-oriented assessment. Artificial seawater exposure, multi-salt solutions, cyclic wetting–drying tests, and long-term performance evaluation are all needed to determine whether an apparent improvement truly persists when chloride, sulfate, magnesium, and leaching act together. This distinction is particularly important for durability evaluation. While single-ion tests are useful for mechanistic isolation, they may overestimate or underestimate actual marine durability because they do not reproduce sulfate–chloride competition, magnesium-induced decalcification, or the coupled release of bound chloride observed under more realistic seawater conditions [7,18].

This issue is also methodological. Bernal et al. [20] and Hu et al. [22] both pointed out limitations of RCPT for directly characterizing chloride diffusion in AAMs. More realistic transport metrics, microstructural characterization, and long-term property retention need to be combined if mitigation strategies are to be compared on a meaningful basis.

### 6.4. Toward Integrated Mitigation Strategies

No single mitigation strategy is universally sufficient for all AAM systems under all marine conditions. Composition and phase design may strengthen chloride immobilization, but this benefit can be offset by strength penalties or long-term instability of calcium-bearing phases. Pore-refinement strategies may delay ion ingress, yet their effectiveness may diminish once cracking, decalcification, or magnesium-driven phase transformation alters the original matrix. Accordingly, the most reliable mitigation approaches are likely to be those that balance chemical resistance, transport control, and phase stability, and that are validated under realistic multi-ion marine exposure rather than under simplified short-term laboratory conditions. This also implies that strategy selection should be system-specific, depending on precursor chemistry, activator type, target exposure condition, and required service life.

To clarify why single-ion evidence cannot be directly extrapolated to realistic marine exposure, Table 5 compares the mechanistic value and limitations of representative single-ion and multi-ion exposure studies. Single-ion chloride, sulfate, or magnesium tests are useful because they isolate specific processes, such as chloride binding and transport, sulfate-induced phase alteration, or magnesium-driven alkalinity loss. However, these tests cannot fully reproduce the competitive and coupled reactions that occur when Cl^−^, SO_4_^2−^, and Mg^2+^ coexist in seawater. Therefore, the purpose of Table 5 is not to rank individual studies, but to show how different exposure designs contribute different levels of mechanistic understanding.

As summarized in Table 5, single-ion tests remain necessary for identifying the dominant role of each aggressive ion, but their interpretation has clear boundaries. Chloride-only exposure mainly reveals transport and binding behavior, sulfate-only exposure helps identify phase alteration and pore-filling/cracking tendencies, and magnesium-related exposure highlights alkalinity reduction and gel destabilization. In contrast, multi-ion exposure is more suitable for revealing interaction effects that cannot be captured by isolated tests, including sulfate–chloride competition, magnesium-induced release of bound chloride, pH reduction, structural breakdown of binding phases, and reopening of transport-effective pores. This comparison explains why the following discussion treats marine degradation as a coupled process rather than as the simple addition of chloride, sulfate, and magnesium effects.

## 7. Macroscopic Durability Evolution of AAMs Under Marine Exposure

The effects of marine ions on AAMs are ultimately significant because they do not remain confined to microscopic interactions. The binding of chloride, the phase alteration induced by sulfate, the destabilization caused by magnesium, and the transport changes governed by pore evolution all progressively manifest at the macroscopic level. Properties such as compressive strength, mass stability, porosity, and water absorption should therefore be interpreted not as isolated indicators, but as macroscopic expressions of underlying chemical and microstructural reconfiguration. In this sense, macroscopic durability evolution should be regarded as the structural expression of preceding chemical and microstructural degradation pathways rather than as a set of independent engineering indicators.

Because the seawater-related durability results summarized in this section were extracted from different published studies, the exposure media and testing procedures were not identical. Some studies used ASTM seawater or artificial seawater, whereas others used natural seawater, seawater curing, seawater corrosion exposure, or saline solutions as related marine-environment simulations. Therefore, these results should not be interpreted as a direct comparison based on one unified seawater recipe or one single testing standard. To clarify the experimental basis of the cited evidence, the exposure media, seawater recreation methods where available, and major evaluation procedures reported in the original studies are summarized in Table 5. As shown in Table 5, the seawater-related evidence used in this section originates from different exposure protocols rather than a single standardized seawater test. Therefore, the following discussion focuses on the reported trends in strength retention, mass change, porosity evolution, pH variation, and microstructural degradation, while interpreting each result within the exposure and testing context of the corresponding original study.

### 7.1. Strength Evolution: From Temporary Densification to Long-Term Degradation

**Figure 9:**
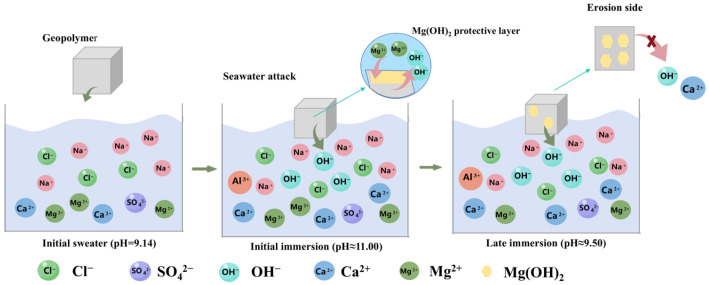
Interaction mechanisms between geopolymer composites and seawater. Prepared by the authors based on mechanisms and experimental observations reported in Ref. [15].

Compressive strength is one of the most widely used durability indicators, yet its evolution under marine exposure is often non-linear. At early ages or during short exposure, continued reaction, secondary precipitation, or local densification may temporarily enhance matrix compactness, leading to stable or even slightly increased strength. At early ages, seawater exposure does not always cause immediate strength loss. Studies on AAFA cementitious materials [54] and alkali-activated metakaolin mortars [55] showed that continued reaction and pore filling can partly offset deterioration. However, longer exposure reveals significant degradation. In metakaolin systems, prolonged seawater immersion leads to strength loss, though quartz powder can mitigate this through filling effects [56]. The addition of slag improves resistance, but brucite precipitation on the surface raises then lowers pH [15]. Under sulfate-containing attack, compressive strength can drop by about 25% due to gypsum formation and C-A-S-H degradation [14]. These quantitative observations indicate that strength evolution under marine exposure is governed by the competition between early-stage densification and long-term structural destabilization. Figure 9 presents the schematic illustration of the interaction mechanisms between geopolymer composites and seawater, which provides a visual link between seawater-induced chemical reactions and macroscopic durability evolution. It shows that seawater exposure may simultaneously induce surface precipitation, pore filling, leaching, and gel alteration. These competing processes explain why some systems exhibit short-term strength retention or pore refinement, while longer exposure may still lead to chemical imbalance and deterioration.

### 7.2. Mass Change and Dimensional Stability

Mass variation records the net outcome of material loss and material accumulation within the exposed matrix. A relatively stable mass may indicate a temporary balance between precipitation and dissolution, but it does not necessarily imply the absence of internal deterioration. Continuous mass loss often signals that dissolution, salt crystallization, or surface damage has become dominant. Tome et al. [57] reported strength and mass-related degradation of volcanic ash–MSWI fly ash alkali-activated mortars after 56 days of artificial seawater exposure, with XRD, FTIR, and SEM used to examine structural and microstructural changes. Zhang et al. [58] should be interpreted as a related saline-environment case rather than a direct artificial-seawater study, because the specimens were immersed in deionized water, salty water, and acidic water, and were evaluated using UCT, static monolithic leaching tests, and ICP-AES analysis. These results illustrate that marine-induced degradation can already be quantitatively measurable at early stages. In Jin et al. [16], the tannery sludge/metakaolin-based geopolymer was subjected to a 21-day seawater corrosion test, after which the residual compressive strength reached 32.64 MPa; XRD, FTIR, and SEM were further used to analyze the microstructural changes. This result indicates that measurable degradation may coexist with retained structural performance under seawater-related exposure. These representative results show that mass evolution is a case-dependent indicator of internal instability. Continuous mass loss may reflect dissolution or surface damage, whereas limited mass loss together with retained strength indicates that measurable degradation can coexist with partial preservation of structural function.

### 7.3. Porosity and Water Absorption: The Microstructural Gateway to Durability

Porosity and water absorption provide a direct link between microstructure and marine durability because they reflect how easily seawater species can penetrate and redistribute within AAMs. Early in marine exposure, pore filling by newly formed products or continued reaction may reduce apparent porosity and water uptake, giving the impression of improved compactness. Over longer periods, however, these beneficial effects may be offset or reversed by decomposition of reaction products, coarsening of transport-effective pores, and loss of continuity in the solid skeleton.

Puertas et al. [17] reported that seawater exposure reduced porosity of AAM mortars by about 17% relative to freshwater exposure, which they linked to the participation of seawater-derived Ca^2+^ and Na^+^ in further gel formation. This result quantitatively illustrates that marine exposure does not always produce immediate pore coarsening; in some systems, short-term densification and long-term deterioration may coexist and should therefore be interpreted dynamically rather than as mutually exclusive trends. More generally, sulfate- and calcium-bearing precipitates may partially fill pore spaces and reduce water absorption, whereas magnesium-induced degradation can eventually coarsen the pore system and increase transport susceptibility. Thus, porosity and water absorption must be interpreted dynamically rather than as static descriptors.

### 7.4. SEM-Based Evidence of Geopolymer Microstructural Evolution

SEM observations provide direct visual evidence for linking marine chemical attack with changes in geopolymer structure. In relatively intact AAM matrices, SEM images commonly show unreacted precursor particles embedded in continuous C-(A)-S-H-, N-A-S-H-, or hybrid gel networks, together with relatively compact pore structures [15,47,57]. After seawater or multi-ion exposure, the observed morphology may change substantially depending on the dominant ion interaction and exposure duration. In some short-term cases, newly formed precipitates or secondary reaction products can fill pores and densify the surface region, which is consistent with temporary strength retention or reduced apparent porosity [15,17]. This type of microstructural refinement helps explain why seawater exposure does not always cause immediate mechanical deterioration.

However, SEM evidence reported in sulfate- and magnesium-containing environments also shows that this initial densification may be unstable. Sulfate attack can be associated with locally accumulated sulfate-bearing products, expansion-related microcracks, and disruption of the original gel skeleton [14,34,35]. Magnesium-rich exposure may produce a more porous and weakened degradation layer because Mg^2+^-induced pH reduction and brucite precipitation promote the destabilization of calcium-bearing gels and the formation of M-S-H/M-A-S-H-type products [7,15,18]. These processes can be manifested in SEM images as pore coarsening, microcracking, loose gel textures, surface scaling, or loss of matrix continuity [16,18,34,57]. Therefore, SEM-based observations support the interpretation that marine degradation of AAMs is not only a chemical process, but also a progressive restructuring of the geopolymer gel network and pore system.

From a durability perspective, the SEM features should be interpreted together with phase and transport indicators. Pore filling or dense surface precipitation may temporarily reduce transport, whereas microcracks, porous alteration layers, and discontinuous gel networks can reopen transport pathways and accelerate further ion ingress [7,18,34]. Thus, SEM images are particularly useful for explaining why strength, mass change, porosity, and chloride transport may evolve nonlinearly during marine exposure [15,16,17,57].

### 7.5. Linking Macroscopic Indicators to Macroscopic Durability and Service-Life Prediction

Phase destabilization, dissolution–precipitation imbalance, and pore-structure reorganization are eventually reflected in measurable durability indicators. Strength retention reflects whether the load-bearing gel network remains continuous; mass change records the balance between dissolution, leaching, precipitation, and surface damage; and porosity or water absorption indicates whether transport resistance is being preserved or lost. Therefore, macroscopic durability indicators should not be interpreted as isolated test results. Their significance depends on whether they are consistent with the underlying phase and microstructural evolution.

To make this microstructure–property linkage more explicit, Table 6 summarizes representative examples in which reported chemical or microstructural changes are connected to measurable macroscopic durability indicators. These examples are not intended to provide a universal ranking of AAM systems, because the cited studies used different binders, exposure media, and test durations. Instead, the table illustrates how specific internal processes, such as brucite precipitation, sulfate-induced phase damage, dissolution–precipitation imbalance, and pore filling, can be reflected in strength retention, strength loss, mass change, or porosity evolution. As shown in Table 6, the same macroscopic indicator may reflect different underlying mechanisms. Strength retention may result from continued reaction, surface precipitation, or temporary pore filling, whereas strength loss may indicate phase degradation, cracking, or loss of gel continuity. Similarly, reduced porosity may reflect short-term densification rather than permanent durability improvement. Therefore, strength, mass change, porosity, and water absorption should be interpreted together with phase analysis and microstructural evidence, rather than being used as stand-alone durability criteria.

Recent modeling studies further strengthen the micro-to-macro linkage discussed above. For example, time-dependent numerical prediction of chloride diffusion in AAFS concretes has been used to estimate chloride profiles and corrosion initiation time, showing that selected AAFS systems may outperform OPC concrete in delaying chloride ingress and corrosion initiation [12]. Similar model-based work on alkali-activated fly ash/slag concretes has shown that slag content and water-to-binder ratio are dominant factors controlling chloride penetration and corrosion initiation time of embedded steel bars, mainly through their effects on porosity and transport resistance [59]. Beyond matrix-level indicators, recent marine durability studies on reinforcement systems also show that microstructural and environmental degradation may ultimately be reflected in reinforcement performance. For instance, marine exposure has been shown to alter the surface morphology and tensile strength retention of GFRP bars embedded in seawater sea–sand concrete, further highlighting the engineering importance of linking internal evolution to serviceability [60]. These case-based observations also indicate that, despite recent progress, major questions remain regarding exposure realism, durability prediction, and engineering validation, which motivates the research gaps and future directions discussed in the next section.

From an engineering-design perspective, the microstructure–property linkage discussed above can be translated into service-life-related indicators such as apparent chloride diffusion coefficient, chloride profile, chloride-binding capacity, corrosion initiation time, strength retention, porosity, and water absorption. For reinforced marine structures, pore refinement and stable chloride binding may delay chloride accumulation at the reinforcement depth, whereas phase alteration, pore coarsening, cracking, or release of bound chloride may increase the effective transport coefficient and shorten the predicted corrosion initiation period. Therefore, microstructural indicators should not be interpreted only as material-characterization results, but also as input parameters for durability design and service-life prediction. Existing durability design concepts for concrete structures, such as exposure classification, cover depth, chloride threshold, diffusion-based prediction, and corrosion initiation criteria, provide a useful engineering framework; however, AAM-specific parameters and standardized multi-ion exposure data are still needed before these concepts can be reliably applied to marine AAM structures.

### 7.6. Implications for Marine Structural Design and Reinforcement Durability

Existing durability design provisions for marine reinforced concrete provide useful reference points for AAM concretes, although they are mainly calibrated for OPC-based systems. In EN 206/BS 8500-type frameworks, marine chloride exposure is commonly divided into exposure classes such as airborne sea-salt exposure, permanent seawater immersion, and tidal/splash-zone exposure. These classes are used together with requirements on minimum cover depth, mixture permeability, strength class, and chloride ingress resistance to reduce the risk of reinforcement corrosion. In service-life-oriented design, chloride-induced corrosion is usually assessed by considering surface chloride concentration, apparent chloride diffusion coefficient, aging factor, cover depth, and chloride threshold at the reinforcement depth [61,62]. Therefore, increased reinforcement cover is not simply a geometric requirement; it acts by extending the chloride transport distance and delaying the time required for chloride concentration at the reinforcement surface to reach the corrosion threshold.

For AAM concretes, this design logic is conceptually applicable but cannot be directly adopted without recalibration. The reason is that the parameters controlling corrosion initiation may differ substantially from OPC systems. AAMs may exhibit different pore solution alkalinity, chloride binding and release behavior, redox environment, phase assemblage, and steel passivation characteristics [7,9,11,18,63,64]. For example, an increased cover depth would be beneficial only if the AAM matrix maintains a low effective chloride transport coefficient and stable chloride immobilization over time. Under seawater multi-ion interactions, sulfate competition and magnesium-induced C-(A)-S-H destabilization may release previously bound chloride and reopen transport-effective pores [7,18]. In such cases, simply increasing cover depth may delay chloride arrival, but it cannot compensate for unstable binding phases or progressive pore coarsening. Therefore, cover design for marine AAM structures should be linked to AAM-specific chloride diffusion/migration coefficients, chloride-binding capacity, chloride threshold, phase stability, and long-term pore-structure evolution.

The relationship between compressive strength and concrete tightness should also be interpreted specifically. Several AAM studies indicate that denser matrices, refined pore structures, lower critical pore size, and reduced water sorptivity or chloride migration are often accompanied by higher compressive strength [20,21,22]. For example, activator optimization and silicate-modulus adjustment can improve strength while reducing chloride transport in slag/fly ash systems [22], and critical pore size has been shown to be more relevant to chloride transport than total porosity in alkali-activated slag concretes [21]. Therefore, increasing strength through pore refinement may improve concrete tightness and delay chloride ingress. However, strength alone is not a sufficient design index for marine AAMs. A high-strength matrix may still suffer from shrinkage-induced microcracking, Mg^2+^ induced gel decomposition, release of bound chloride, or loss of alkalinity. Thus, concrete tightness should be evaluated using combined indicators, including compressive strength, water absorption, sorptivity, electrical resistivity, chloride migration/diffusion coefficient, pore-size distribution, and free/bound chloride ratio.

Regarding reinforcement durability, corrosion tests have been performed on steel embedded in or exposed to alkali-activated systems, but the available evidence remains less mature than for OPC concrete. Studies on steel corrosion in AAMs show that passivation and chloride-induced depassivation can differ markedly from Portland-cement systems, because the onset of pitting and the chloride threshold depend strongly on pore solution alkalinity and redox conditions [63]. Recent reviews also emphasize that chloride ingress, chloride binding, and corrosion rates of steel reinforcement in AAMs are not yet sufficiently understood and require long-term laboratory and field studies [64]. Therefore, future corrosion assessment of reinforced AAM concrete should include half-cell potential, corrosion current density, linear polarization resistance, electrochemical impedance spectroscopy, chloride threshold determination, and bond-performance evaluation under marine wetting–drying or immersion conditions.

Non-metallic reinforcement provides another possible route for marine structures. FRP and GFRP bars do not undergo conventional electrochemical corrosion like steel, which makes them attractive for chloride-rich environments. However, they are not automatically durability-free solutions. Their long-term performance may be affected by highly alkaline pore solutions, seawater salinity, temperature, sustained stress, fiber/matrix interface degradation, and concrete cover thickness. Recent marine concrete research on GFRP bars embedded in seawater sea–sand concrete showed that exposure time, temperature, seawater salinity, and cover thickness can influence surface morphology and tensile strength retention [60]. For AAM concretes, similar studies are still needed because AAM pore solutions may contain high concentrations of OH^−^, alkalis, silicate, and aluminate species, which may interact differently with FRP/GFRP surfaces. Therefore, both metallic and non-metallic reinforcement should be evaluated in future marine AAM studies, and the design of reinforced AAM structures should couple matrix durability with reinforcement passivation, bond durability, and long-term structural serviceability.

## 8. Research Gaps and Future Directions for the Marine Durability of AAMs

Although substantial progress has been made in understanding the marine durability of AAMs, current knowledge remains fragmented and unevenly distributed across mechanisms, material systems, and exposure conditions. Existing studies have separately addressed broad durability, chloride transport, and seawater-ion effects, but a unified framework that simultaneously links the main marine-ion threats, the intrinsic material basis of different AAM systems, and the mitigation strategies developed to preserve durability is still lacking.

Based on the literature reviewed above, the existing research gaps can be highlighted in several aspects. First, the coupled effects of Cl^−^, SO_4_^2−^, and Mg^2+^ remain insufficiently quantified, and many conclusions are still derived from single-ion or simplified exposure tests. Second, the relationship between precursor chemistry, phase assemblage, pore-structure evolution, and long-term marine durability has not yet been established on a common comparative basis. Third, the stability of chloride-binding phases under realistic multi-ion exposure remains uncertain, especially when sulfate competition, magnesium-induced alkalinity loss, and leaching occur simultaneously. Fourth, standardized testing protocols for artificial seawater, ion concentration ranges, wetting–drying cycles, and long-term exposure duration are still lacking. Finally, engineering-level validation remains limited, particularly for reinforced AAM concretes, chloride threshold determination, corrosion initiation, and field or near-field service performance.

Future work should therefore move beyond general durability evaluation and focus on several actionable priorities. First, benchmark multi-ion exposure protocols should be established to reproduce realistic Cl^−^-SO_4_^2−^-Mg^2+^ interactions under controlled artificial seawater, multi-salt, and cyclic wetting–drying conditions. Second, coupled transport-reaction models should be developed to quantitatively link chloride ingress, chloride binding and release, sulfate-related phase alteration, magnesium-induced alkalinity loss, pore-structure evolution, and service-life indicators such as chloride profiles and corrosion initiation time. Third, comparative studies should be conducted on representative high-calcium, low-calcium, and hybrid AAM systems under identical exposure protocols, so that the effects of precursor chemistry and activator design can be evaluated on a common basis. Fourth, long-term field or near-field validation should be prioritized in splash, tidal, and immersion zones, where leaching, oxygen availability, wetting–drying cycles, and ion accumulation differ substantially from simplified laboratory immersion tests.

In particular, future research should extend from paste, mortar, and plain-concrete matrices to reinforced AAM concrete. For steel-reinforced AAM concretes, key tests should include chloride threshold determination, corrosion initiation time, half-cell potential, corrosion current density, linear polarization resistance, electrochemical impedance spectroscopy, steel-AAM bond strength, and residual mechanical performance after marine exposure. These tests should be conducted under artificial seawater, multi-salt exposure multi-salt solutions, wetting–drying cycles, and long-term field or near-field exposure. In addition, FRP/GFRP-reinforced AAM concretes should be evaluated in terms of tensile strength retention, bond durability, surface morphology, interfacial damage, and sustained-load performance, because non-metallic reinforcement can avoid conventional electrochemical corrosion but may still be affected by alkaline pore solutions and marine exposure. Such reinforced-specimen studies are essential for translating matrix-level durability findings into structural-level serviceability and design guidance.

## 9. Conclusions

This review reinterprets the marine durability of alkali-activated materials (AAMs) from a coupled multi-ion perspective rather than from a chloride-only or single-ion viewpoint. By integrating literature evidence on Cl^−^, SO_4_^2−^, and Mg^2+^ attack, this review clarifies how marine ions progressively weaken the intrinsic protective functions of AAMs through transport, binding competition, pore solution evolution, phase destabilization, and microstructural reorganization. The following conclusions and implications can be drawn.

(1) Marine multi-ion exposure affects AAMs through coupled rather than isolated processes. Chloride mainly governs binding and transport, sulfate modifies phase relations and chloride-related behavior, and magnesium most strongly destabilizes the internal alkaline and structural equilibrium. Their combined action progressively transforms an initially protective matrix into a more vulnerable one.
(2) The large variability reported in the literature is primarily rooted in material differences rather than simple inconsistency among studies. High-calcium, low-calcium, and hybrid AAM systems differ markedly in phase assemblage, pore solution chemistry, chloride-binding behavior, and phase stability, which explains their divergent durability responses under similar marine exposure conditions.
(3) Many apparently conflicting observations, such as short-term densification versus long-term deterioration, can be rationalized by considering exposure stage, calcium availability, binding-phase stability, and the evolution of transport-effective pore structure. This highlights the need to interpret marine durability dynamically rather than through isolated indicators or single exposure stages.
(4) Future progress requires a shift from descriptive mechanism studies toward design-oriented durability frameworks. Priority tasks include establishing standardized multi-ion exposure protocols, developing coupled transport-reaction models linked to service-life indicators, conducting benchmark comparisons among representative AAM categories, performing long-term field validation under realistic marine conditions, and assessing chloride threshold, corrosion initiation, and reinforcement compatibility in reinforced AAM concretes.

Overall, the present review newly clarifies that the marine durability of AAMs should be understood as a coupled multi-ion degradation process rather than as a chloride-only problem. The key unresolved issues include the lack of standardized multi-ion exposure protocols, insufficient quantitative models linking ion transport, phase evolution, pore-structure change, and service-life indicators, and limited long-term field validation of reinforced AAM systems under realistic marine conditions. From an engineering perspective, these findings imply that the design of marine AAMs should move beyond single-property optimization and instead balance chloride-binding capacity, transport resistance, phase stability, and exposure-relevant validation for specific service environments.

## Figures and Tables

**Table 1:** Evaluation basis used for interpreting the degradation information summarized in Section 3.

Evaluation Aspect	Typical Information Extracted from Literature	Purpose in Section 3
Chloride-related behavior	Chloride diffusion/migration coefficient, passed charge, free/bound chloride content, chloride-binding isotherm	To evaluate chloride ingress, binding, and transport resistance
Sulfate-related behavior	Sulfate immersion, sulfate–chloride combined exposure, expansion, strength loss, gypsum/ettringite formation	To evaluate sulfate-induced competition, phase alteration, and cracking tendency
Magnesium-related behavior	MgCl_2_/MgSO_4_/seawater exposure, pH evolution, brucite precipitation, C-(A)-S-H decomposition, M-S-H formation	To evaluate alkalinity reduction and phase alteration
Multi-ion coupling	Artificial seawater or chloride–sulfate–magnesium multi-salt exposure	To evaluate whether ion effects are additive, competitive, or mutually amplified
Macroscopic durability	Compressive strength, mass change, dimensional stability, porosity, water absorption	To connect chemical/microstructural degradation with engineering performance
Microstructural/chemical evidence	XRD, SEM/EDS, FTIR, TG/DTG, MIP, pore solution analysis	To identify phase transformation, pore evolution, and degradation pathways

**Table 2:** Critical comparison between representative single-ion and multi-ion exposure studies on marine durability of AAMs.

Exposure Type	What It Clarifies	Main Limitation	Representative Studies
Single-ion chloride exposure	Chloride binding, free transport, phase-dependent resistance	Cannot reflect sulfate competition or magnesium-induced chloride release	Jiang et al. [32]; Jun et al. [37]
Single-ion sulfate exposure	Phase alteration, pore filling, cracking tendency, cation-dependent effects	Cannot represent chloride-related transport under realistic seawater chemistry	Shu et al. [35]; Coppola et al. [38]
Multi-ion chloride–sulfate-magnesium exposure	Coupled effects on binding, pH, phase stability, chloride release, pore connectivity	More realistic but also more complex, requiring system-specific interpretation	Chen and Ye [7]; Liu et al. [18]

**Table 3:** Categorization of AAM systems based on precursor calcium content and their representative marine-durability characteristics.

Category	Representative Precursors	Dominant Reaction Products	Main Chloride-Binding Mode	Typical Marine Durability Implication
High-calcium systems	Slag-based AAMs (AAS)	C-(A)-S-H, LDH/hydrotalcite, AFm/Friedel’s salt-related phases	Chemical binding + gel adsorption	Usually strong chloride immobilization and relatively low diffusion, but calcium-bearing phases may be vulnerable to Mg^2+^-induced destabilization
Low-calcium systems	Fly ash- and metakaolin-based AAMs	N-A-S-H	Mainly physical adsorption	Often weaker chemical chloride fixation and more transport-sensitive behavior
Hybrid systems	Slag/fly ash blends; slag/metakaolin blends	Mixed C-(A)-S-H/N-A-S-H/(C,N)-A-S-H	Mixed mechanism, often adsorption-dominated at high low-calcium content	Potential to combine advantages of both systems, but performance is highly ratio-dependent

**Table 4:** Bound chloride content of waterglass-activated (WAS) and NaOH-activated slag (NAS) at varying pH values [12].

pH Value	Amount of Bound Chloride (mg/g-Sample)	
	WAS	NAS
11.0	37.83	34.95
11.5	35.36	30.53
12.0	30.76	27.49
12.5	30.01	23.01
13	28.11	19.29
13.5	25.95	18.71

**Table 5:** Comparative evaluation of representative mitigation strategies against marine-ion threats in AAMs.

Strategy Category	Typical Approach	Main Benefit	Main Trade-Off/Limitation	Long-Term Marine Applicability
Composition/phase design	Ca-bearing phase promotion	Higher chloride immobilization	May reduce strength; calcium-rich phases may be vulnerable to Mg^2+^ attack	Promising, but needs long-term validation under Mg-containing marine exposure
LDH-based additives	Mg-Al LDH/calcined LDH	Improved chloride binding	Evidence mainly from lab-scale chloride-focused systems	Potentially useful, but insufficient multi-ion field validation
Pore-structure refinement	Higher silicate modulus/optimized alkali dosage	Lower chloride transport, denser matrix	Effect may be offset by later cracking or chemical destabilization	Useful for short-to-medium term, but not sufficient alone
Mineral admixture densification	Microsilica/nanoparticles	Reduced permeability	Nanosilica agglomeration may increase heterogeneity and transport	Highly system-dependent
Exposure-oriented evaluation	Artificial seawater/multi-salt/cyclic tests	More realistic assessment	More complex, less comparable across studies	Essential for practical screening

**Table 6:** Representative examples linking microstructural evolution and macroscopic durability indicators in AAMs under marine exposure.

Representative Literature Example	Internal Evolution	Macroscopic Indicator	Interpretation
Kuang et al. [15]	Brucite precipitation and pH evolution	Strength retention/delayed deterioration	Early surface precipitation may temporarily suppress leaching
Al-Antaki and Niş [14]	Sulfate-induced phase damage	~25% strength loss	Phase degradation is translated into measurable structural weakening
Jin et al. [16]	Dissolution/precipitation balance	3–5% mass loss with 32.64 MPa residual strength	Measurable degradation may coexist with retained function
Puertas et al. [17]	Secondary gel formation/pore filling	~17% porosity reduction	Seawater exposure may induce transient densification

## Data Availability

No new data were created or analyzed in this study. Data sharing is not applicable to this article.
